# Characterization of the complete mitochondrial genome of *Notospermus geniculatus*

**DOI:** 10.1080/23802359.2018.1522976

**Published:** 2018-10-03

**Authors:** Jin-qing Jiang, Rui-guang Deng

**Affiliations:** a College of Animal Science and Veterinary Medicine, Henan Institute of Science and Technology, Xinxiang, Henan Province, China;; b Key Laboratory of Animal Immunology, Henan Academy of Agricultural Sciences, Zhengzhou, China

**Keywords:** *Notospermus geniculatus*, mitochondrial genome, assembly, phylogeny

## Abstract

In this study, the complete mitochondrial genome of *Notospermus geniculatus,* was recovered through Illumina sequencing data. This complete mitochondrial genome of *N. geniculatus* is 15,180 bp in length and has a base composition of A (14.4%), T (41.3%), C (14.6%), G (29.6%), demonstrating a bias of higher AT content (55.7%) than GC content (44.2%). The mitochondrial genome contains a typically conserved structure among Lineidae mitogenomes, encoding 13 protein-coding genes (PCGs), 23 transfer RNA genes (tRNA), 2 ribosomal RNA genes (12S rRNA and 16S rRNA), and a control region (D-loop region). All PCGs were located on the H-strand. ND4L gene and ND4 gene were overlapped by 6 bp. The whole mt genome of *N. geniculatus* and other Protostomia mitogenomes (17 species, in total) were used for phylogenetic analysis. The result indicated *N. geniculatus* has the closest relationship with *Lineus viridis* (FJ839919.1) and clustered within Heteronemertea Clade, which representing a distinct order.

*Notospermus geniculatus* is one of the largest nemerteans present in Mediterranean waters, reaching 1 m in length and 15 mm in width. This species was originally described from Naples and is also widely distributed (Kajihara 2007). Here, we provide a report of the complete mitochondrial genome of *Notospermus geniculatus.*

In this study, a specimen of *N. geniculatuss* was collected from Balearic Islands (38°73′N, 1°41′E) and fixed in absolute ethanol. Genomic DNA was extracted from muscle tissues using DNEasy Extraction Kit (supplied by QIAGEN) following the manufactures instructions. The isolated DNA was stored in the sequencing company (HuiTong Tech, Shenzhen, China). Purified DNA was fragmented and used to construct the sequencing libraries following the instructions of NEBNext^®^ Ultra™ II DNA Library Prep Kit (NEB, BJ, CN). Whole genomic sequencing was performed by the Illumina HiSeq 2500 Sequencing Platform (Illumina, San Diego, CA). Adapters and low-quality reads were removed using the NGS QC Toolkit (Patel et al. 2012). Then assembly as implemented by SPAdes 3.9.0 (Bankevich et al. 2012). Circularization of this mt genome was confirmed using MITObim V1.9 (Hahn et al. 2013). The complete sequence was primarily annotated by ORF prediction in Unipro UGENE (Okonechnikov et al. 2012) combined with manual correction. All tRNAs were confirmed using the tRNAscan-SE search server (Lowe et al. 1997). Other protein-coding genes were verified by BLAST search on the NCBI website (http://blast.ncbi.nlm.nih.gov/), and manual correction for start and stop codons were conducted. This complete mitochondrial genome sequence together with gene annotations were submitted to GenBank under the accession numbers of MH714705.

The complete mitochondrial genome of *Notospermus geniculatus* was 15,180 bp in length and has a base composition of (14.4%), T (41.3%), C (14.6%), G (29.6%), demonstrating a bias of higher AT content (55.7%) than GC content (44.2%). The mitochondrial genome contains a typically conserved structure among Lineidae mitogenomes, encoding 13 protein-coding genes (PCGs), 23 transfer RNA genes (tRNA), 2 ribosomal RNA genes (12S rRNA and 16S rRNA), and a control region (D-loop region).All PCGs were located on the H-strand. ND4L gene and ND4 gene were overlapped by 6 bp.

For phylogenetic analysis assessing the relationship of this mitogenome, we selected other 17 Protostomia mitogenomes from Lineidae (4 taxa), Monostilifera (4 taxa), Palaeonemertea (1 taxon), Molluscae (4 taxa), and Ecdysozoa (2 taxa) to construct genome-wide alignment. The genome-wide alignment of all mt genomes was done by HomBlocks (Bi et al. 2018) under trimAl method, which containing all phylogenetic informative gaps in alignments, resulting in 4248 positions in total, including three conserved fragments shared by all mitogenomes. The whole genome alignment was analyzed by IQ-TREE version 1.6.6 (Nguyen et al. 2014) under the TIM3 + F + R3 model. The tree topology was verified under both 1000 bootstrap and 1000 replicates of SH-aLRT test. The resulting tree was represented and edited using FigTree v1.4.1. As shown in Figure 1, the phylogenetic positions of these 17 mt genomes were successfully resolved with high bootstrap and SH-aLRT supports except with one node. The result indicated *N. geniculatus* has the closest relationship with *Lineus viridis* (FJ839919.1) and clustered within Heteronemertea Clade, which represents a distinct order.

**Figure 1.**
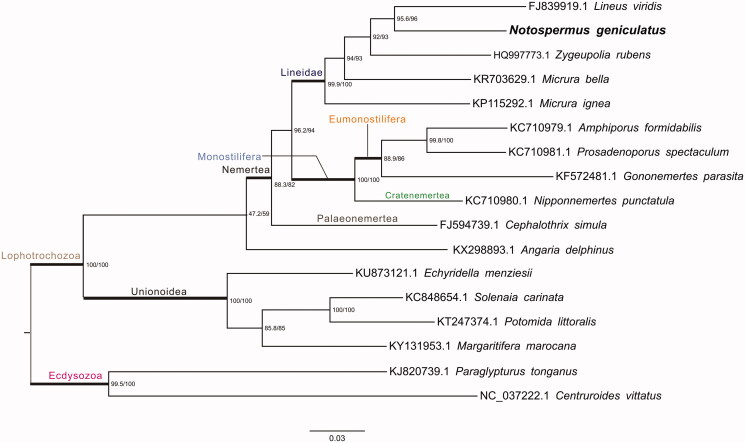
Phylogenetic tree yielded by IQ-TREE of 17 Protostomia mitogenomes. Consensus tree is shown with support indicated by numbers at branches, representing percentages of SH-aLRT test and bootstraps.
